# Simultaneous Determination of Six Acidic Pesticides, Including 2,4-DB and 2,4,5-T with No Established MRL in Korea Using LC-MS/MS and QuEChERS for the Safety of Imported Agricultural Products

**DOI:** 10.3390/foods14050904

**Published:** 2025-03-06

**Authors:** Joon-Kyung Oh, Jae-Hyeong Kim, Ga-Eul-Hae An, Hee-Ra Chang

**Affiliations:** Department of Pharmaceutical Engineering, Graduate School, Hoseo University, Asan 31499, Republic of Korea; 555wnsrud@naver.com (J.-K.O.); jaebro99@naver.com (J.-H.K.); nofallsun1004@naver.com (G.-E.-H.A.)

**Keywords:** acidic pesticides, pesticide residue, food safety, PLS, monitoring

## Abstract

The use of pesticides is essential for the production of high-quality agricultural products. However, the repeated application of pesticides has led to the contamination of environments, such as the atmosphere, soil, and surface water, affecting ecosystems and leading to residues on crops, which pose potential risks to human health. Accordingly, regulations regarding pesticide usage, application frequency, timing, and maximum residue limits have been established to manage residual pesticides. The Positive List System (PLS), with a default tolerance of 0.01 ppm, has been applied to both domestic and imported agricultural products for which no established maximum residue limits (MRLs) exist in Korea. This study developed a multi-residue analytical method for the simultaneous determination of six pesticides, including 2,4-DB and 2,4,5-T, for which no domestic MRLs have been established, as well as for 2,4-D, 4-CPA, Dicamba, and Dichlorprop, for the management of imported agricultural products. The target pesticides were extracted and purified using the QuEChERS method and quantified by LC-MS/MS. The analytical method was validated according to the CODEX (CAC/GL 40-1993) and the Guidelines of Standard Procedures of Test Methods for Foods and Other Substances established by the MFDS. Using the developed and validated analytical method, monitoring of imported agricultural products was conducted.

## 1. Introduction

Pesticides are primarily aimed at controlling weeds during the cultivation of agricultural products and protecting crops from pests to prevent post-harvest damage [1,2,3,4]. These plant protection products are essential for securing the quality of agricultural products. However, the application of pesticides often results in their unintentional dispersion into the air, soil, and water, thereby disrupting ecosystems. Their misuse or overuse may lead to the emergence of pesticide-resistant pests. Furthermore, residual pesticides that penetrate crops or adhere to their surfaces pose risks to consumers by accumulating in their bodies and potentially causing toxicity, thus threatening human health [5,6]. This issue is ongoing, and each country has implemented safety management practices for residual pesticides through various policies, such as establishing maximum residue limits (MRLs) for domestic and imported agricultural products and setting Pre-Harvest Intervals (PHIs) concerning the amount, timing, and frequency of pesticide application [7,8].

In 1995, the establishment of the World Trade Organization (WTO) led to increased trade activity between countries. Since then, the signing of Free Trade Agreements (FTAs) between Korea and other countries has expanded [9,10,11], resulting in a rise in the importation of agricultural products [11,12]. Korea’s primary import partners are the United States, the European Union (EU), the ASEAN, China, and Australia. In 2022, the import value of agricultural products increased by 17.7% compared to the previous year [13]. As consumer demand for imported agricultural products increases, concerns regarding residual pesticides are also rising. Consequently, ensuring the safety of residual pesticides has become more important. Additionally, the types of and directions for the use of pesticides, along with their MRLs, may be unestablished or vary between countries due to different environmental conditions and the pests encountered in agricultural cultivation [14,15]. In Korea, for imported agricultural products, pesticide residues are verified using the analytical methods specified in the Korean Food Code during the import customs clearance process to conduct a safety evaluation. Since January 2019, the Ministry of Food and Drug Safety (MFDS) has used the Positive List System (PLS), which has been implemented in the EU and Japan. The PLS applies to pesticide residues that do not have an established MRL in Korea, and these residues are managed by applying a default MRL of 0.01 mg/kg [15,16,17,18]. Through this system, the misuse and abuse of pesticides used domestically can be prevented, and the importing and distribution of non-compliant agricultural products from other countries can be blocked. It is especially necessary to secure a quick and reliable residual analysis method that can detect levels of 0.01 mg/kg of pesticides without established MRLs in Korea among imported agricultural products.

Phenoxy carboxylic acid (PCA), including 2,4-DB and 2,4,5-T, used as an herbicide to control broad leaf weed, have established MRLs in Japan, the EU, and Australia, but MRLs have not been set in Korea. Furthermore, there is no analytical method for the agricultural commodities listed in the Food Code for determining the residues of these pesticides on imported agricultural products into Korea. The analytical methods for the acidic pesticides (2,4-D, Dicamba, 4-CPA, and Dichlorprop) on agricultural commodities, as reported previously, use gas chromatography (GC) and high-performance liquid chromatography (HPLC), following solvent extraction and SPE clean-up [19,20,21,22,23]. Earlier reports on these herbicides [24,25] proposed mobile-phase compositions. In our study, we aimed to reduce the retention time of the analytes and, consequently, shorten the total run time. Furthermore, we employed 0.1% formic acid in both the aqueous (water) and organic (methanol) phases during gradient elution. Maintaining a consistent pH throughout the gradient improves the peak shapes and reproducibility. We chose not to use ammonium formate due to its potential for ion suppression in an LC-MS analysis, which could negatively impact the sensitivity. Ammonium formate may interact with residual silanol groups in the column, leading to peak broadening or tailing, particularly for acidic herbicides [26]. However, these methods are time-consuming and complex for determining the selectivity, sensitivity, and reproducibility. Recently, simultaneous analytical methods for pesticides using the QuEChERS (Quick, Easy, Cheap, Effective, Rugged, and Safe) method for a quick and precise analysis by LC-MS/MS have been employed. This research aims to establish a reliable quantitative and qualitative method for the simultaneous determination of six acidic pesticides, including 2,4-DB and 2,4,5-T, as PCA group herbicides, to ensure the safety of imported agricultural products.

## 2. Materials and Methods

### 2.1. Selection of Agricultural Products

For the optimization and validation of analytical methods, five representative agricultural products (cereal grains—hulled rice, root and tuber vegetables—potato, legume vegetables—soybean, fruits—mandarin, and fruiting vegetables—green pepper) were selected considering the Codex food category system and the priory list of domestic food consumption. These commodities were purchased as pesticide-free organic agricultural products from hypermarkets.

A total of 54 imported agricultural products were purchased online and offline for monitoring purposes. These included 19 fruits (3 oranges, 3 cherries, 2 lemons, 1 rainbow mango, 1 pineapple, 1 blueberry, 1 black sapphire grape, 1 melon, 1 lime, 1 red grape, 1 green grape, 1 raspberry, 1 kiwi, and 1 banana), 10 grains (3 quinoas, 2 Kamuts, 1 Calrose rice, 1 corn, 1 millet, 1 farro, and 1 oat groat), 13 vegetables (3 green peppers, 1 red pepper, 1 garlic scape, 1 spinach, 1 green onion, 1 red bell pepper, 1 okra, 1 leek, 1 broccolini, 1 shallot, and 1 asparagus), 10 legumes (3 soybeans, 2 lentils, 2 chickpeas, 1 kidney bean, 1 white kidney bean, and 1 adzuki bean), and 2 tubers (potato). The purchased samples were cultivated in various countries, including the United States, China, Australia, Vietnam, Peru, Chile, Thailand, New Zealand, the Philippines, Canada, Italy, Belgium, and Spain.

All were stored in a deep freezer for over 24 h, then homogenized using a blender, and subsequently stored in a freezer prior to analysis.

### 2.2. Reagents and Chemicals

UHPLC-grade organic solvent Acetonitrile (ACN) and methanol (MeOH) were purchased from J.T.Baker^®^ (Avantor Performance Materials Korea Ltd., Suwon-Si, Republic of Korea). Formic acid (HPLC grade: purity, 99%) for use in the mobile phase was purchased from FUJIFILM (Wako Pure Chemical Corp., Osaka, Japan). A QuEChERS extraction kit (the EN Method No. 5982-7650), a dispersive solid-phase extraction (d-SPE), a Primary Secondary Amine (PSA, No. 5982-5021), and C_18_ (No. 5982-4921 and No. 5982-4956) were purchased from Agilent (Agilent Technologies Korea Ltd., Seoul, Republic of Korea). Pesticide analytical standards for 2,4-DB, 2,4,5-T, 4-CPA, Dicamba, and Dichlorprop were purchased from HPC Standards GmbH (Cunnersdorf, Germany), and 2,4-D was purchased from SIGMA Aldrich (St. Louis, MI, USA). The physicochemical properties of these pesticides are shown in Table A1.

A mixed standard solution was prepared by aliquoting each pesticide from a 100 mg/L standard solution to achieve a concentration of 10 μg/mL. This solution was then diluted stepwise with 90% matrix extract to concentrations of 0.002, 0.0045, 0.01, 0.02, 0.035, and 0.05 μg/mL to prepare a matrix-matched standard solution for a calibration curve.

### 2.3. Instrumental Conditions for Analysis

An AB SCIEX ExionLC^TM^ Series UHPLC (AB SCIEX, Concord, ON, Canada), connected to an AB SCIEX QTRAP 5500 LC-MS/MS system (AB SCIEX, Concord, ON, Canada) was adopted for the simultaneous quantification of six pesticides in agricultural products. The mobile phase used to detect target analytes consisted of 0.1% formic acid in water (A) and 0.1% formic acid in methanol (B). Gradient elution was performed under the following conditions: 90% solvent A and 10% solvent B from 0.0 to 1.0 min, transitioning to 20% A and 80% B from 4.0 to 5.0 min, and then returning to 90% A and 10% B at 6.0 min, which was maintained until 10.0 min. The flow rate was set at 0.3 mL/min. A reversed-phase Unison UK-C_18_ column (100 × 2 mm, 3 μm, Imtakt, Kyoto, Japan), effective for separating hydrophilic and hydrophobic compounds, was used to separate the analytes from interference. The column temperature was set to 35 °C, and the injection volume was 3 μL. Considering the tendency of acidic pesticides to ionize by losing a proton and forming conjugate bases, we employed the negative mode of electrospray ionization (ESI) [26]. Nitrogen was used as the collision gas. The conditions of the mass spectrometry source were set as follows: curtain gas (CUR) at 30.0 psi, collision gas (CAD) set to medium, ion spray voltage (IS) at −4500.0 V, source temperature (TEM) at 550 °C, ion source gas 1 (GS1) at 60.0 psi, and ion source gas 2 (GS2) at 50.0 psi. Quantitative analysis was conducted in Multi Reaction Monitoring (MRM) mode to enhance selectivity and sensitivity for the analytes by selecting two precursor ions and their corresponding product ions based on their excellent intensity. The product ion demonstrating the highest sensitivity was selected as the quantifier ion, while the ion with the second highest sensitivity was used as the qualifier ion. Detailed values for the precursor ions and product ions, along with the collision energies for the analytes, are listed in Table A2.

### 2.4. Optimization of Quantitation Method

Initially, soybean and mandarin were used to optimize the analytical method. This selection was based on the protein and fat composition of soybeans [27], and the high levels of acidic amino acids, organic sugars, and vitamins in mandarins [28], which may be co-extracted with target pesticides as interfering substances. Five grams of the prepared samples were weighed and placed into 50 mL conical tubes. For soybeans, which were in a dried state, 5 mL of distilled water was added and allowed to hydrate for 30 min to increase extraction efficiency. The samples were treated with a pesticide mixture standard solution at fortified levels of 0.01, 0.1, and 0.5 μg/g to conduct the recovery test (*n* = 3). After the addition of 10 mL of acetonitrile, the samples were subjected to shaking extraction at 1300 rpm using a Genogrinder (Lab system, Seoul, Republic of Korea) for 1 min. Subsequently, a QuEChERS extraction using the citrate-buffered method was performed by adding the EN kit, which contained 0.5 g of Na_2_HCit·1.5H_2_O, 1 g of Na_3_Cit·2H_2_O, 1 g of NaCl, and 4 g of MgSO_4_. The samples were shaken for 1 min and then centrifuged at 4000 rpm for 5 min using a centrifuge (Fleta 5, Hanil Science, Seoul, Republic of Korea). Three different clean-up methods (PSA—Method 1, C_18_—Method 2, and C_18_—Method 3) were compared to evaluate the efficiency of interference removal. A supernatant (1 mL for Methods 1 and 2, and 5 mL for Method 3) was taken and purified in a d-SPE tube, and the purified aliquot was analyzed by LC-MS/MS after filtration using a syringe filter (0.45 μm, PTFE, Korea vaccine, Seoul, Republic of Korea).

### 2.5. Validation of Analytical Method

The analytical method was validated for selectivity, linearity, limit of detection, limit of quantification, accuracy, and precision based on CODEX (CAC/GL 40-1993) [29] and the Guidelines for Standard Procedures for Test Methods for Foods and Other Substances issued by the Ministry of Food and Drug Safety (MFDS, 0116-01, April 2016) [30]. The method parameters were obtained using Microsoft Excel 2016 (Microsoft, Redmond, WA, USA). Selectivity was confirmed by comparing the chromatograms of the matrix-matched standard solution (0.0045 μg/mL) and matrix solution to determine whether there were any interference peaks at the retention time of the analytes. Additionally, according to the SANTE/11312/2021 guidelines [31], the relative intensities of the quantifier and qualifier ions were compared with those of the reference standard to confirm that they fell within an acceptable tolerance of ±30%. The retention time of the detected compound was required to be within ±0.1 min of the reference standard to ensure proper identification [32]. The limit of detection (LOD) and limit of quantitation (LOQ) were calculated by analyzing the standard solutions at concentrations of 0.002, 0.0045, 0.01, 0.02, 0.035, and 0.05 μg/mL, measured in triplicate to construct the calibration curve. The calculations for LOD and LOQ were based on the average slope of the calibration curve and the standard deviation of the response values. The LOQ was determined through analysis using LC-MS/MS, ensuring that the signal-to-noise ratio (S/N) was greater than 10 based on the calculated LOQ. The method limit of quantitation (MLOQ) was calculated to be 0.01 mg/kg, considering the limit of quantitation (LOQ, S/N ≥ 10), final volume, and sample weight [33]. Linearity was evaluated by measuring the matrix-matched standard solution at concentrations ranging from 0.002 to 0.05 μg/mL to plot the calibration curve. Linearity was validated by obtaining a coefficient of determination within the acceptance criteria (r^2^ ≥ 0.98). The accuracy and precision were determined by analyzing each pesticide in blank samples of five agricultural products, which were spiked at three concentration levels: the MLOQ (0.01 mg/kg), 10 times the MLOQ (0.1 mg/kg), and 50 times the MLOQ (0.5 mg/kg), with five replicates for each level. Recovery and coefficient of variation (CV, %) for each concentration were evaluated, confirming that the results were within the acceptance criteria.

Matrix effect (ME, %) refers to the enhancement or suppression of ionization resulting from interactions between analytes and interfering compounds during mass spectrometry analysis [34,35]. To effectively account for matrix influence when analyzing pesticides, analytes were quantified using matrix-matched calibration curves. The calibration curve was constructed based on the peak area of a standard solution and the matrix-matched standard solution was prepared by mixing 90% of the blank sample extract with the standard solution. Matrix effects were calculated using the slopes of the calibration curve and the matrix-matched calibration curve using the following Equation (1).

(1) $$\mathrm{Matrix}\;\mathrm{effect}\;(\mathrm{ME},\;\%)=\left(\frac{\mathrm{Slope}\;\mathrm{of}\;\mathrm{matrix}-\mathrm{matched}\;\mathrm{calibration}\;\mathrm{curve}}{\mathrm{Slope}\;\mathrm{of}\;\mathrm{solvent}\;\mathrm{calibration}\;\mathrm{curve}}-1\right)\times 100$$

A positive matrix effect value indicates increased ion intensity (ion enhancement), while a negative matrix effect value indicates decreased ion intensity (ion suppression). The matrix effect was categorized into three ranges as follows: (1)|ME| < 20% was considered soft, (2) 20% ≤ |ME| < 50% was considered medium, and (3)|ME| > 50% was considered strong [36].

The ion ratio of each pesticide was calculated as the ratio of quantifier to qualifier ion intensities for analyte identification. The reference ion ratio was determined as the mean value of the ion ratio obtained from six different concentrations (0.002, 0.0045, 0.01, 0.02, 0.035, and 0.05 μg/mL) of matrix-matched standard solutions. The ion ratio of the sample was calculated as the mean ion ratio of the spiked samples at the MLOQ level (0.01 mg/kg, *n* = 5). The relative tolerance was verified by comparing the ion intensities of the reference and sample ion ratios in accordance with the 2002/657/EC guidelines and calculated using the following Equation (2) [37].

(2) $$\mathrm{Relative}\;\mathrm{tolerance}\;(\%)=\left(\frac{\mathrm{Ion}\text{\_}\mathrm{ratio}\left(sample\right)-\mathrm{Ion}\text{\_}\mathrm{ratio}\left(Ref\right)}{\mathrm{Ion}\text{\_}\mathrm{ratio}\left(Ref\right)}\right)\times 100$$

### 2.6. Pesticide Monitoring of Imported Agricultural Products Using the Validated Method

The validated method, including sample preparation, was applied to 54 samples of imported agricultural products collected online for monitoring purposes, consisting of 19 fruits (3 oranges, 3 cherries, 2 lemons, 1 rainbow mango, 1 pineapple, 1 blueberry, 1 black sapphire grape, 1 melon, 1 lime, 1 red grape, 1 green grape, 1 raspberry, 1 kiwi, and 1 banana), 10 grains (3 quinoas, 2 Kamuts, 1 Calrose rice, 1 corn, 1 millet, 1 farro, and 1 oat groat), 13 vegetables (3 green peppers, 1 red pepper, 1 garlic scape, 1 spinach, 1 green onion, 1 red bell pepper, 1 okra, 1 leek, 1 broccolini, 1 shallot, and 1 asparagus), 10 legumes (3 soybeans, 2 lentils, 2 chickpeas, 1 kidney bean, 1 white kidney bean, and 1 adzuki bean), and 2 tubers (potato), originating from the United States, China, Australia, Vietnam, Peru, Chile, Thailand, New Zealand, the Philippines, Canada, Italy, Belgium, and Spain. Online sales through platforms significantly outpaced offline sales, a shift attributed to increased flexibility in scheduling, competitive pricing, and delivery convenience following COVID-19 [38].

Quantitation was performed by matrix-matched calibration curves for each representative agricultural product, and recovery samples at the MLOQ level (0.01 mg/kg) were analyzed in triplicate simultaneously for quality control.

## 3. Results and Discussion

### 3.1. Optimization and Verification of the QuEChERS Method

**Figure 1:**
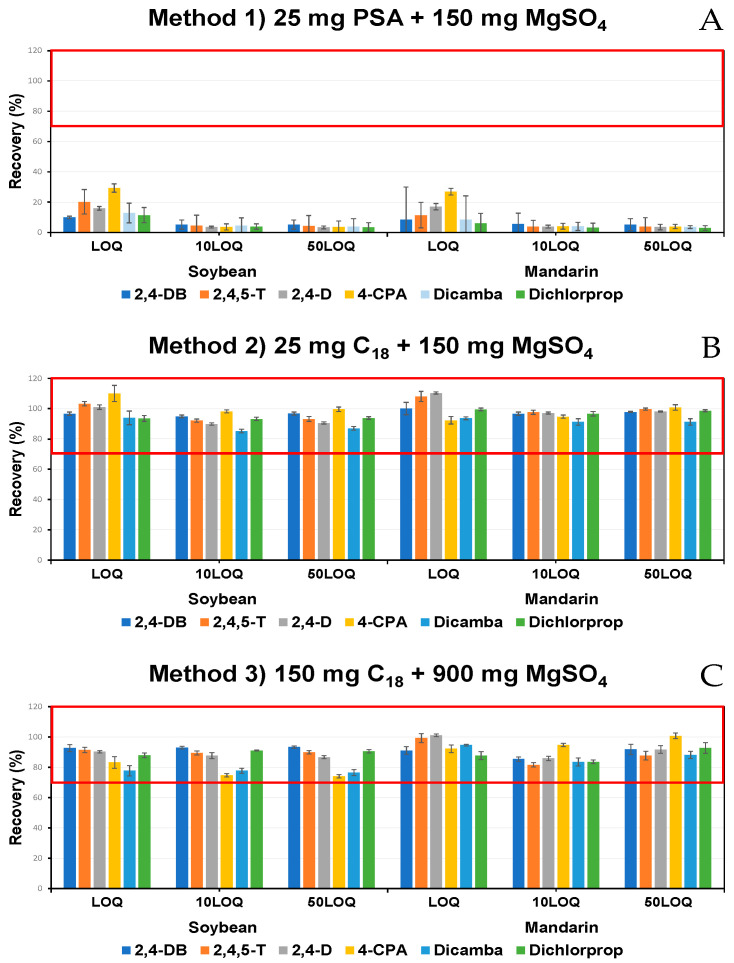
Recoveries according to adsorbents. (**A**) Method 1: 25 mg PSA + 150 mg MgSO_4_. (**B**) Method 2: 25 mg C_18_ + 150 mg MgSO_4_. (**C**) Method 3: 150 mg C_18_ + 900 mg MgSO_4_.

The residue definitions of PCA, including 2,4-DB, 2,4,5-T, 2,4-D, and Dichlorprop, are described as the sum of their salt, ester, and conjugate forms. PCA herbicides are often applied in ester formulations. When pesticides are applied in ester formulations, they become more lipophilic, which enhances their penetration into crop tissues. While some esters can be detected in harvested crops, most undergo rapid hydrolysis and conjugation within a plant, converting them into free acids and conjugates. As a result, in harvested crops, pesticides predominantly exist in their free acid form rather than as esters. Based on this understanding, we initially designed our analytical method specifically for the quantification of free acid forms [39]. The extraction of acidic pesticides in agricultural products was difficult due to their polar characteristics and high water solubility. Acetonitrile was used as the extraction solvent, and citrate buffer was added to minimize ionization and subsequently enhance the partitioning of the analytes into the acetonitrile phase [40]. In the purification step, the purification efficiency of two adsorbents (PSA and C_18_) was compared. The results of the purification using PSA (Method 1) showed a low recovery of 2.9% to 29.3% (Figure 1A). The low recovery of acidic pesticides was due to their adsorption onto the PSA, which resulted from its anion exchange properties [41,42].

**Figure 2:**
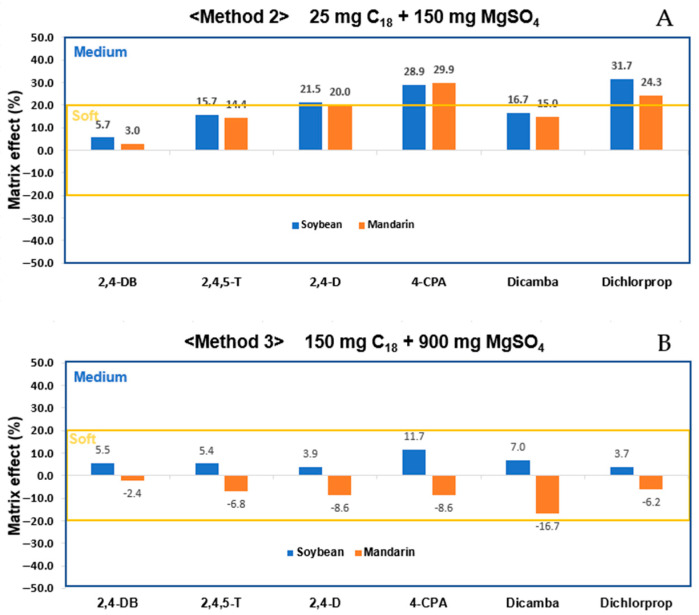
Comparison of matrix effect of (**A**) Method 2: 25 mg C_18_ + 150 mg MgSO_4_; (**B**) Method 3: 150 mg C_18_ + 900 mg MgSO_4_.

For the purification using C_18_, the recovery and the percentage of the coefficient of variation (%CV) for Method 2 (25 mg C_18_ + 150 mg MgSO_4_) were 85.2% to 110.3%, with a %CV of 5.3% (Figure 1B). For Method 3 (150 mg C_18_ + 900 mg MgSO_4_), the recovery was 74.0% to 101.1%, with a %CV of 3.8% (Figure 1C). Both methods met the acceptable criteria (LOQ: 60–120%, ±32%; 10LOQ: 70–120%, ±22%; 50LOQ: 70–110%, ±18%) specified in the guidelines. However, a comparative analysis of the matrix effect showed that Method 2 had a range of 3.0% to 31.7% (Figure 2A), while Method 3 exhibited a range of −16.7% to 11.7% (Figure 2B). Method 2 demonstrated soft levels of matrix effect, whereas Method 3 displayed effects ranging from soft to medium [43].

The analytical method was optimized by using acetonitrile in the extraction step with the citrate method (EN kit) to partition the analytes from the agricultural products, and the adsorbent from Method 3 (150 mg C_18_ and 900 mg MgSO_4_), which exhibited a soft matrix effect, was utilized in the clean-up process. The optimized analytical method was validated to verify the validation parameters.

### 3.2. Method Validation

#### 3.2.1. Selectivity, LOD, and LOQ

**Figure A1:**
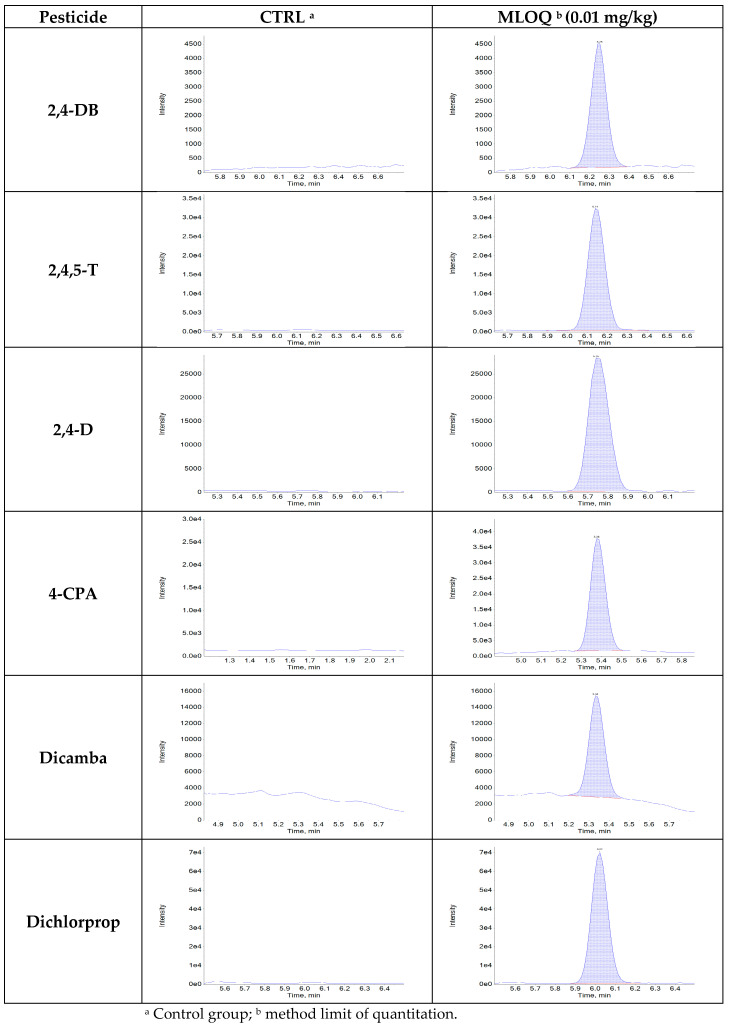
Representative chromatograms of the samples (CTRL and MLOQ) for the analytes.

The selectivity was confirmed by the absence of interfering peaks, which was determined by comparing the chromatograms of the matrix solution and the matrix-matched standard solution (0.0045 μg/mL) for each pesticide (Figure A1). All six analytes met the criteria, with the relative intensities of the quantifier and qualifier ions falling within the acceptable tolerance of ±30% compared to the reference standard at the MLOQ (2,4-DB, −11.2~0.6; 2,4,5-T, −11.1~14.4; 2,4-D, −1.0~5.2; 4-CPA, −11.7~0.9; Dicamba, −1.0~0.6; Dichlorprop, −10.6~6.2). Additionally, the retention times of the detected compounds were within ±0.1 min of the reference standard, ensuring their proper identification (2,4-DB, 6.24~6.26 min; 2,4,5-T, 6.14~6.16 min; 2,4-D, 5.74~5.77 min; 4-CPA, 5.37~5.39 min; Dicamba, 5.33~5.35 min; Dichlorprop, 6.01~6.04 min). The limit of detection (LOD) and limit of quantitation (LOQ) were calculated from the standard deviation of the response (y-intercept) and slope of the calibration curves at concentrations of 0.002, 0.0045, 0.01, 0.02, 0.035, and 0.05 μg/mL, and were measured in triplicate (Table 1) [44].

The LOQ was confirmed by analyzing the standard solution at 0.0045 μg/mL, which exceeded the calculated LOQ, using HPLC-MS/MS. This analysis verified that the signal-to-noise (S/N) ratio was greater than 10. The MLOQs of the pesticides were determined at a level of 0.01 mg/kg, which is the default MRL for monitoring imported agricultural products and satisfies the requirements of the Positive List System in Korea.

#### 3.2.2. Linearity

The matrix-matched calibration curves were plotted for each pesticide at six concentration levels (0.002, 0.0045, 0.01, 0.02, 0.035, and 0.05 μg/mL), and the coefficient of determination (r^2^) was calculated from the linear regression equations to evaluate the linearity. The coefficient of determination (r^2^) was higher than 0.99 for every pesticide, confirming that it was within the acceptable linear range (Table 2).

#### 3.2.3. Accuracy and Precision

The accuracy and precision were determined by analyzing each pesticide in blank samples of five agricultural products, which were spiked at three concentration levels: the MLOQ, 10 times the MLOQ, and 50 times the MLOQ, with five replicates for each level. The results indicated that the mean recovery and percentage coefficient of variation (%CV) of the analytes across the five agricultural products met the acceptable accuracy and precision for the respective concentration levels (LOQ: 60–120%, ±32%; 10x LOQ: 70–120%, ±22%; 50x LOQ: 70–110%, ±18%) in accordance with the CODEX guidelines (Table 3).

#### 3.2.4. Matrix Effect

The results of the matrix effect analysis indicated that for 2,4-DB, the matrix effect ranged from −7.1% to 10.2%; for 2,4,5-T, it ranged from −12.5% to 7.7%; for 2,4-D, it ranged from −18.3% to 10.6%; for 4-CPA, it ranged from 0.1% to 14.6%; for Dicamba, it ranged from −26.2% to 14.0%; and for Dichlorprop, it ranged from −16.8% to 15.9%. With the exception of Dicamba in brown rice (medium), all the matrix effect values were classified within the soft range. In the cases of soft matrix effects, the analytical method demonstrated excellent purification efficiency, with minimal influence from sample–analyte interactions, indicating that quantification can be performed using a standard calibration curve rather than a matrix-matched calibration curve [45].

#### 3.2.5. Ion Ratio

The ion ratio was calculated from the peak intensities of the quantifier and qualifier MRM transition of the analytes in both the matrix-matched standard solution and the sample extract for confirmation purposes. The relative tolerance was determined to compare the ion ratios, expressed as a percentage, of the matrix-matched standard solution and sample extract, to verify the variations in ion ratios. The relative tolerance range was divided into four categories based on the ion ratio: (1) ion ratio ≤ 10%, ±50%; (2) 10% < ion ratio ≤ 20%, ±30%; (3) 20% < ion ratio ≤ 50%, ±25%; and (4) 50% < ion ratio, ±20%. The calculated ion ratios for the analytes were as follows: 2,4-DB, 11.6–16.0%; 2,4,5-T, 58.5–63.5%; 2,4-D, 8.6–8.8%; 4-CPA, 12.6–13.9%; Dicamba, 33.2–33.4%; and Dichlorprop, 7.3–7.9%. The relative tolerance criteria based on the ion ratios were ±30% for 2,4-DB and 4-CPA; ±20% for 2,4,5-T; ±50% for 2,4-D and Dichlorprop; and ±25% for Dicamba, according to the 2002/657/EC guidelines [36]. As shown in Table 4, the ion ratios of all the pesticides in all the sample matrices were within the acceptable tolerance criteria, indicating the accuracy of the quantification mass.

#### 3.2.6. Pesticide Monitoring of Imported Agricultural Products

The analysis of imported agricultural products was conducted using the validated method to quantify the residues of six pesticides. The recovery range and %CV of the quality control samples were from 77.2% to 111.4% and 9.0%, respectively, both of which were within the acceptable ranges of 60–120% and ±32% [30]. All the real sample quantifications were performed using matrix-matched calibration curves of the representative crops, which were selected to reflect the characteristics of each crop group (e.g., legumes, grains, fruits, fruiting vegetables, and tuber vegetables).

2,4-D was detected in one orange sample from the USA at an average concentration of 0.014 mg/kg (*n* = 3). It is commonly used as a plant growth regulator that enhances invertase activity and promotes sugar accumulation, thereby improving fruit sweetness and quality and increasing fruit size [46]. In Korea, the MRL of 2,4-D for citrus fruits is set at 0.15 mg/kg, and the detected residue level of 0.014 mg/kg is below the MRL, indicating safety for consumers. The residues of the other samples for the six pesticides, for which no MRLs were established, were either undetected or below the MLOQ of 0.01 mg/kg (Table 5). These residue levels are lower than the default MRL (0.01 mg/kg), indicating that they are legally acceptable and safe for public health.

## 4. Conclusions

The import of agricultural products is steadily increasing, resulting in a growing number of cases where pesticide residues are detected. While some residual pesticides may persist from their application during cultivation, others may be detected when pesticides are used for long-term storage or to enhance quality prior to export. To manage pesticide residues, national regulations have established MRLs for pesticides in agricultural products. Since the implementation of the PLS system in Korea, pesticides without an established MRL in agricultural products have been managed according to a default MRL (≤0.01 mg/kg). This study optimized and validated the simultaneous QuEChERS method for six acidic pesticides, including 2,4-DB and 2,4,5-T, for which MRLs have been established in Korea, enabling the rapid and reliable quantitation of imported products. The validation parameters for selectivity, linearity, the limit of detection, the limit of quantification, accuracy, and precision met the acceptable criteria based on the CODEX guidelines (CAC/GL 40-1993) and the Guidelines for Standard Test Methods for Foods and Other Substances (April 2016) established by the MFDS (0116-01). The residues of pesticides in 54 imported agricultural products were analyzed to verify the application of the proposed method. It was determined that for 53 samples, pesticide residues were either not detected or were below the limit of quantification, while 2,4-D was detected in one sample (orange) at a level below the MRL established in Korea, indicating safety for public health. Therefore, this simultaneous analytical method for six acidic pesticides will be useful for monitoring the pesticide residues in and ensuring the safety management of imported agricultural products.

## Figures and Tables

**Table 1:** The standard solution calibration curve’s linear regression equation, coefficient of determination, LOD, and LOQ for six pesticides.

Pesticide	Linear Regression		LOD ^a^ (mg/kg)	LOQ ^b^ (mg/kg)
	Equation	r^2^		
2,4-DB	y = 7990819.42x − 9504.70	0.9958	0.0011	0.0037
2,4,5-T	y = 7748012.3914x − 8054.70	0.9989	0.0005	0.0016
2,4-D	y = 38900297.02x − 38136.35	0.9984	0.0004	0.0015
4-CPA	y = 42920484.90x − 45643.87	0.9987	0.0008	0.0025
Dicamba	y = 14478819.18x − 1441.53	0.9997	0.0002	0.0007
Dichlorprop	y = 55988246.52x + 6841.23	0.9987	0.0011	0.0037

^a^ Limit of detection; ^b^ Limit of quantitation.

**Table 2:** Linearity of matrix-matched calibration curve by matrix for target analytes.

Pesticide	r^2^ (Coefficient of Determination)				
	Soybean	Mandarin	Hulled Rice	Green Pepper	Potato
2,4-DB	0.9989	0.9989	0.9994	0.9991	0.9994
2,4,5-T	0.9997	0.9993	0.9984	0.9994	0.9983
2,4-D	0.9994	0.9990	0.9995	0.9990	0.9990
4-CPA	0.9978	0.9984	0.9956	0.9956	0.9989
Dicamba	0.9996	0.9996	0.9998	0.9991	0.9992
Dichlorprop	0.9997	0.9994	0.9999	0.9998	0.9996

**Table 3:** Recovery and coefficient of variation for six pesticides on five agricultural products.

Analyte	MLOQ ^a^ (mg/kg)	Fortified Level (mg/kg)	Recovery (%), *n* = 5									
			Soybean		Mandarin		Hulled Rice		Green Pepper		Potato	
			Mean ± SD ^b^	%CV ^c^	Mean ± SD	%CV	Mean ± SD	%CV	Mean ± SD	%CV	Mean ± SD	%CV
2,4-DB	0.01	0.01	92.7 ± 2.2	2.4	91.1 ± 2.4	2.6	85.4 ± 3.0	3.5	91.8 ± 2.3	2.5	102.8 ± 3.4	3.3
		0.1	93.0 ± 0.8	0.9	85.4 ± 1.3	1.5	87.1 ± 2.0	2.3	92.9 ± 0.4	0.4	98.6 ± 0.9	0.9
		0.5	93.3 ± 0.7	0.8	91.8 ± 3.2	3.5	80.9 ± 1.8	2.2	94.2 ± 1.4	1.5	100.3 ± 0.2	0.2
2,4,5-T		0.01	91.4 ± 1.6	1.7	99.3 ± 3.0	3.0	94.1 ± 2.0	2.1	104.6 ± 1.4	1.3	109.4 ± 4.5	4.1
		0.1	88.8 ± 1.2	1.3	82.3 ± 1.2	1.4	83.1 ± 1.3	1.6	92.3 ± 0.6	0.7	98.4 ± 0.7	0.7
		0.5	89.9 ± 1.0	1.1	87.7 ± 2.5	2.9	80.2 ± 0.8	1.0	93.3 ± 1.8	1.9	99.0 ± 1.0	1.0
2,4-D		0.01	90.3 ± 0.7	0.8	101.1 ± 0.9	0.9	88.5 ± 1.3	1.4	106.5 ± 0.8	0.7	113.6 ± 3.2	2.8
		0.1	86.9 ± 1.7	2.0	86.7 ± 1.3	1.5	82.1 ± 1.4	1.7	94.4 ± 0.6	0.6	100.7 ± 1.0	1.0
		0.5	86.7 ± 0.8	0.9	91.7 ± 2.3	2.5	79.8 ± 1.1	1.3	94.8 ± 1.3	1.3	102.6 ± 0.6	0.6
4-CPA		0.01	83.2 ± 3.1	3.8	92.3 ± 2.3	2.5	95.7 ± 2.1	2.2	97.4 ± 2.5	2.5	114.5 ± 4.4	3.9
		0.1	74.1 ± 0.8	1.1	95.3 ± 1.0	1.1	78.7 ± 1.5	1.9	94.3 ± 0.5	0.5	96.8 ± 2.0	2.1
		0.5	74.0 ± 0.8	1.1	100.8 ± 1.8	1.8	75.6 ± 1.1	1.4	94.8 ± 1.6	1.7	99.4 ± 1.7	1.7
Dicamba		0.01	77.7 ± 2.7	3.4	94.7 ± 0.6	0.6	79.1 ± 3.2	4.0	97.8 ± 2.7	2.7	104.8 ± 1.6	1.5
		0.1	78.2 ± 1.2	1.6	84.5 ± 2.2	2.6	86.1 ± 1.5	1.8	94.2 ± 1.3	1.4	102.5 ± 1.5	1.5
		0.5	76.5 ± 1.5	2.0	88.2 ± 2.1	2.4	89.3 ± 0.9	1.0	94.0 ± 0.9	1.0	101.3 ± 2.4	2.4
Dichlorprop		0.01	88.0 ± 1.4	1.6	87.7 ± 2.2	2.6	87.3 ± 1.7	2.0	95.6 ± 1.4	1.5	108.3 ± 2.9	2.6
		0.1	91.1 ± 0.3	0.4	84.2 ± 1.1	1.3	83.7 ± 1.7	2.0	94.3 ± 0.4	0.4	98.4 ± 0.9	0.9
		0.5	90.6 ± 0.9	1.0	92.8 ± 3.2	3.5	81.0 ± 0.7	0.9	95.1 ± 1.0	1.0	99.1 ± 0.9	0.9

^a^ The method limit of quantitation; ^b^ standard deviation; ^c^ percentage coefficient of variation, Standard deviation/Mean × 100.

**Table 4:** Ion ratio and relative tolerance of six acidic analytes.

Analyte	Soybean		Mandarin		Hulled Rice		Green Pepper		Potato	
	Ion Ratio (%) ^a^	Relative Tolerance (%) ^b^	Ion Ratio (%)	Relative Tolerance (%)	Ion Ratio (%)	Relative Tolerance (%)	Ion Ratio (%)	Relative Tolerance (%)	Ion Ratio (%)	Relative Tolerance (%)
2,4-DB	14.3	−8.7	13.1	−1.9	16.0	0.6	16.0	−7.0	11.6	−11.2
2,4,5-T	63.5	1.4	58.5	−11.1	62.0	−3.2	61.2	14.4	61.4	3.0
2,4-D	8.7	5.2	8.8	−1.0	8.6	0.4	8.6	2.2	8.8	4.8
4-CPA	13.4	−1.7	12.6	0.5	13.3	0.9	13.7	−11.7	13.9	-1.8
Dicamba	33.3	−1.0	33.4	0.0	33.2	0.6	33.4	−0.2	33.4	0.4
Dichlorprop	7.7	6.2	7.9	−10.6	7.3	2.7	7.5	0.9	7.8	−7.6

^a^ Peak area_(qualifier)_/Peak area_(quantifier)_ × 100; ^b^ (Ion_ratio_(sample)_ − Ion_ratio_(Ref)_)/Ion_ratio_(Ref)_ × 100.

**Table 5:** Pesticide residues in imported samples were analyzed according to the validated method.

Commodity Class	Sample	Origin	Residue Amount (μg/g) (Mean/%CV ^a^)					
			2,4-DB	2,4,5-T	2,4-D	4-CPA	Dicamba	Dichlorprop
Legumes	Soybean	China	ND ^b^	ND	ND	ND	ND	ND
	Soybean	China	<0.01	ND	ND	ND	ND	ND
	Soybean	China	ND	ND	ND	ND	ND	ND
	Chickpea	Canada	ND	ND	ND	ND	ND	ND
	Kidney bean	China	ND	ND	ND	ND	ND	ND
	Lentil	Canada	ND	ND	ND	ND	ND	ND
	White kidney bean	China	ND	ND	ND	ND	ND	ND
	Adzuki bean	China	ND	ND	ND	ND	ND	ND
	Lentil	Canada	ND	ND	ND	ND	ND	ND
	Chickpea	Canada	ND	ND	ND	ND	ND	ND
Fruits	Orange	USA	<0.01	ND	<0.01	ND	ND	ND
	Orange	USA	ND	ND	0.014/3.6	ND	ND	ND
	Orange	Australia	<0.01	ND	<0.01	ND	ND	<0.01
	Cherry	USA	<0.01	ND	<0.01	ND	ND	ND
	Cherry	USA	<0.01	ND	<0.01	ND	<0.01	ND
	Cherry	USA	ND	ND	<0.01	ND	<0.01	ND
	Rainbow mango	Thailand	ND	ND	ND	ND	ND	ND
	Lemon	Chile	ND	ND	ND	ND	ND	ND
	Pineapple	The Philippines	ND	ND	ND	ND	ND	ND
	Blueberry	USA	ND	ND	ND	ND	ND	ND
	Black sapphire grape	USA	ND	ND	ND	ND	ND	ND
	Melon	USA	ND	ND	ND	ND	ND	ND
	Lemon	USA	ND	ND	ND	ND	ND	ND
	Lime	Vietnam	ND	ND	ND	ND	ND	ND
	Red grape	USA	ND	ND	ND	ND	ND	ND
	Green grape	USA	ND	ND	ND	ND	ND	ND
	Raspberry	Chile	ND	ND	ND	ND	ND	ND
	Kiwi	New Zealand	ND	ND	ND	ND	ND	ND
	Banana	The Philippines	ND	ND	ND	ND	ND	ND
Grains	Quinoa	USA	ND	ND	<0.01	ND	ND	<0.01
	Quinoa	USA	ND	ND	<0.01	ND	ND	<0.01
	Quinoa	Peru	ND	ND	<0.01	ND	ND	<0.01
	Calrose rice	USA	<0.01	ND	ND	ND	ND	<0.01
	Corn	USA	ND	ND	ND	ND	ND	<0.01
	Kamut	Canada	ND	ND	ND	ND	ND	ND
	Millet	China	ND	ND	ND	ND	ND	ND
	Farro	Italy	ND	ND	ND	ND	ND	ND
	Oat groat	Canada	ND	ND	ND	ND	ND	ND
	Kamut	Canada	ND	ND	ND	ND	ND	ND
Tuber Vegetables	Potato	USA	ND	ND	ND	ND	ND	ND
	Potato	USA	ND	ND	ND	ND	ND	ND
Fruiting Vegetables	Green Pepper ^c^	China	ND	ND	ND	ND	ND	ND
	Green Pepper ^c^	China	ND	ND	<0.01	ND	ND	ND
	Green Pepper ^c^	China	ND	ND	ND	ND	ND	ND
	Red Pepper ^c^	Vietnam	ND	ND	ND	ND	ND	ND
	Garlic scape	China	ND	ND	ND	ND	ND	ND
	Spinach	China	ND	ND	ND	ND	ND	ND
	Green onion	China	ND	ND	ND	ND	ND	ND
	Red bell pepper	China	ND	ND	ND	ND	ND	ND
	Okra	China	ND	ND	ND	ND	ND	ND
	Leek	Belgium	ND	ND	ND	ND	ND	ND
	Broccolini	Spain	ND	ND	ND	ND	ND	ND
	Shallot	Australia	ND	ND	ND	ND	ND	ND
	Asparagus	New Zealand	ND	ND	ND	ND	ND	ND

^a^ Percentage coefficient of variation, standard deviation/Mean × 100 (*n* = 3); ^b^ not detected; ^c^ frozen state.

## Data Availability

The original contributions presented in this study are included in the article. Further inquiries can be directed to the corresponding author.

## Appendix A

**Table A1:** Physicochemical properties and chemical structure of targeted acidic pesticides.

Properties	Acidic Pesticides					
	2,4-DB	2,4,5-T	2,4-D	4-CPA	Dicamba	Dichlorprop
Chemical Structure						
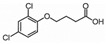
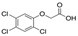
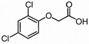
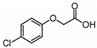
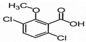
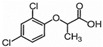
Classification	Herbicide	Herbicide	Herbicide	PGR ^d^	Herbicide	PGR, Herbicide
Group	PCA ^e^	PCA	PCA	PCA	Benzoic acid	PCA
M.f. ^a^	C_10_H_10_Cl_2_O_3_	C_8_H_5_Cl_3_O_3_	C_8_H_6_Cl_2_O_3_	C_8_H_7_ClO_3_	C_8_H_6_Cl_2_O_3_	C_9_H_8_Cl_2_O_3_
Mol. Wt. ^b^	249.1	255.5	221.0	186.6	221.0	235.1
Log K_ow_	−0.25 (pH 9) 1.35 (pH 7) 2.94 (pH 5)	4.0	−1.07 (pH 10) −0.82 (pH 7) 1.54 (pH 4)	2.52	−1.9 (pH 8.9) −1.88 (pH 6.8) −0.55 (pH 5.0)	<1.0 (pH 7, 9) 1.11 (pH 5)
pKa (20−25 °C)	4.1	2.88	3.4	3.18	1.87	3.0
V.p. ^c^	0.0944 (23.6 °C)	1 × 10^−7^ (25 °C)	0.0099 (20 °C) 0.023 (25 °C)	0.024 (25 °C)	1.67 (25 °C, calc.)	<0.01 (20 °C)
Water solubility (mg/l, 20−25 °C)	62.0 (pH 5) 4385.0 (pH 7) 4.548 × 10^5^ (pH 9)	268 (25 °C)	3390.0 (pH 4) 2.0031 × 10^4^ (pH 5) 2.43 × 10^4^ (pH 7) 2.65 × 10^4^ (pH 10)	Insoluble	6600.0 (pH 1.8) >2.5 × 10^5^ (pH 4.1, 6.8, 8.2)	350.0

^a^ Molecular formula; ^b^ molecular weight (g/mol); ^c^ vapor pressure (mPa); ^d^ plant growth regulator; ^e^ phenoxy carboxylic acid.

**Table A2:** Multi-reaction monitoring conditions for quantitation of pesticides.

Pesticide	Exact Mass	Ionization Mode	MRM Transitions						T_R_^e^
			Precursor Ion, *m/z*	Product Ion, *m/z*	DP ^a^	EP ^b^	CE ^c^	CXP ^d^	
2,4-DB *	248.0	[M−H]^−^	247.0	160.8	−64	−10	−12	−10	6.25
2,4-DB-1			248.6	162.9	−35	−10	−18	−18	
2,4,5-T *	253.9	[M−H]^−^	253.0	159.0	−50	−10	−40	−22	6.15
2,4,5-T-1			253.0	195.0	−50	−10	−40	−22	
2,4-D *	220.0	[M−H]^−^	219.0	160.9	−19	−10	−14	−10	5.75
2,4-D-1			219.0	124.9	−10	−10	−34	−26	
4-CPA *	186.0	[M−H]^−^	185.0	126.8	−71	−10	−18	−10	5.38
4-CPA-1			186.7	128.9	−40	−10	−20	−18	
Dicamba *	220.0	[M−H]^−^	219.0	175.0	−21	−10	−6	−12	5.34
Dicamba-1			218.9	35.0	−30	−10	−35	−18	
Dichlorprop *	234.0	[M−H]^−^	233.0	161.0	−26	−10	−14	−12	6.03
Dichlorprop-1			233.0	125.1	−26	−10	−34	−26	

^a^ Declustering potential; ^b^ entrance potential; ^c^ collision Energy; ^d^ collision exit potential; ^e^ retention time; * quantifier ion.
